# Plasma Homocysteine Is Associated with Aortic Arterial Stiffness but not Wave Reflection in Chinese Hypertensive Subjects

**DOI:** 10.1371/journal.pone.0085938

**Published:** 2014-01-27

**Authors:** Wenkai Xiao, Yongyi Bai, Ping Ye, Leiming Luo, Dejun Liu, Hongmei Wu, Jie Bai

**Affiliations:** 1 Department of Geriatric Cardiology, Chinese PLA General Hospital, Beijing, China; 2 Department of Clinical Biochemistry, Chinese PLA General Hospital, Beijing, China; Idaho State University, United States of America

## Abstract

**Objective:**

Elevated plasma total homocysteine (tHcy) acts synergistically with hypertension to exert a multiplicative effect on cardiovascular diseases risk. The aim of this study was to determine the relationship between tHcy concentration and blood pressure, and to evaluate the role of plasma tHcy in arterial stiffness and wave reflection in hypertension.

**Methods:**

In this cross-sectional study, a community-based sample of 1680 subjects (mean age 61.6 years) was classified into four groups according to tHcy level (<21.6 vs. ≥21.6 µmol/l) and blood pressure (hypertensive vs. normotensive). Levels of plasma tHcy and other biochemical parameters (e.g., lipids, glucose) were determined. Central arterial blood pressure, reflected pressure wave, and carotid-femoral pulse wave velocity (cf-PWV) were assessed by tonometry within 2 days of obtaining the blood specimen.

**Results:**

Neither peripheral nor central blood pressure differed according to tHcy levels in normotensive and hypertensive subjects. Differences in cf-PWV according to tHcy were observed only in hypertensive subjects; differences in cf-PWV in normotensive subjects were not significant after adjusting for confounding factors. Central augmentation index did not differ according to tHcy level in either normotensive or hypertensive subjects. Results of univariate analysis revealed significant correlations between blood pressure parameters and tHcy concentration only among normotensive subjects; however, these correlations were not significant in a partial correlation analysis. Results of multiple regression analysis showed that plasma tHcy levels were independently correlated with cf-PWV in hypertensive subjects (β = 0.713, P = 0.004). The independent relationship between tHcy and central augmentation index was not significant by further multiple analyses in normotensive or hypertensive individuals.

**Conclusions:**

Plasma tHcy level is strongly and independently correlated with arterial stiffness measured as cf-PWV only in hypertensive subjects. Thus, hypertension is a major link between tHcy and aortic arterial stiffness.

## Introduction

Recent studies have reported that elevated tHcy may be deleterious in individuals with hypertension or other risk factors (e.g., cigarette smoking, hypercholesterolemia), with which it acts synergistically to exert a multiplicative effect on cardiovascular disease (CVD) risk [1]–[2]. In patients with coronary heart disease, those with both hypertension and high tHcy levels had more severe coronary atherosclerosis and more diffuse atherosclerosis than those without this association [3]. This combination of elevated tHcy and hypertension has been described as “H-type hypertension” [4]–[5].

The pathological mechanisms underlying the interaction between hypertension and hyperhomocysteinemia in CVD and cerebrovascular diseases are not fully understood but may include their similar effects on the vascular system or oxidative stress [6]. Arterial stiffness can be detected before the appearance of clinically significant vascular disease, suggesting that it may be a marker for the development of atherosclerotic disease [7] or a causative factor in atherosclerosis [8]–[9]. Although previous studies have reported the association of plasma tHcy with arterial stiffness, those results are controversial because of differences in study populations and methods of assessing arterial stiffness [10]–[11]. Furthermore, few prospective studies have investigated the role of tHcy and hypertension on arterial stiffness in Asian populations [6], which have patterns of cerebrovascular disease and CVD that are distinct from those of Caucasians and African Americans. Therefore, further investigation is needed to clarify the relationship between plasma tHcy and arterial stiffness in hypertension.

The purpose of this study was to investigate the following in a large community-based sample from China: (1) relationship between hypertension complicated by hyperhomocysteinemia with increased arterial stiffness and wave reflection; (2) relationship between tHcy and peripheral, central arterial blood pressure (BP); (3) influence of plasma tHcy and other risk factors on arterial stiffness and wave reflection by measuring pulse wave velocity (PWV) and augmentation index (AIx) in hypertensive and normotensive individuals.

## Methods

### Study Population

This community-based cross-sectional study was carried out in the Pingguoyuan area of Shijingshan district, Beijing, China. A total of 1859 community residents reporting for a health examination in two communities were randomly recruited to the study. We excluded 31 individuals with severe systemic diseases including collagenosis, endocrine and metabolic diseases other than diabetes mellitus (DM), inflammation, neoplastic disease, or severe liver or renal disease. We attempted to assess arterial stiffness in the remaining 1828 subjects; however, adequate tonometry was either not attempted or not obtained in 86 participants. Another 37 participants were excluded because of missing data (plasma tHcy level or other biochemical measurements). An additional 25 participants were excluded because of missing covariate data needed for multivariable analysis. The remaining 1680 participants were eligible for analysis. This study was approved by the ethics committee of People’s Liberation Army General Hospital, and written informed consent was obtained from all participants.

### Clinical Data Collection

All participants were interviewed and completed a standardized questionnaire that included questions about prevalent diseases, family history of CVD, medication use, and lifestyle factors. Physical examinations and interviews were carried out by trained medical doctors. Self-reported smoking status was categorized as current, former, or never. Height and weight were measured in a standing position without shoes. Body mass index was calculated as weight in kilograms divided by the square of height in meters. Peripheral BP was measured two times in the right brachial artery; measurements were taken at 5-min intervals, and the average systolic blood pressure (SBP) and diastolic blood pressure (DBP) measurements were used for analysis and further calculation. Mean arterial blood pressure (MAP) was calculated as DBP+[1/3 × (SBP – DBP)]. Hypertension was defined exclusively by the peripheral BP measurement.

### Biochemical Measurements

All subjects underwent full laboratory evaluation (lipidemic profile, Hepatic and renal function indices). After a 12-h fasting (no alcohol), blood was collected from the antecubital vein between 8 a.m. and 10 a.m., with the subject in a sitting position.

Biochemical variables of all blood specimens were measured with an automated analyzer (Roche Cobas e601, Switzerland) in the same laboratory, following the criteria of the World Health Organization Lipid Reference Laboratories. All participants without a history of DM underwent the standard 75-g oral glucose tolerance test (OGTT). The estimated glomerular filtration rate (eGFR) was calculated using the Chinese modification of diet in renal disease (C-MDRD) equation [12]: eGFR (ml/min/1.73 m^2^) = 175 × standard creatinine (mg/dl)^−1.234^ × age (year)^−0.179^ × (0.79 if female). Plasma tHcy level was determined by high-performance chromatography with fluorometric detection, with a lower limit of detection of 0.5 µmol/l, and inter-assay variation of 4.1%.

### Measurements of Arterial Properties

Measurement of arterial properties was conducted in the morning, in a quiet environment, at a stable temperature. The subjects were asked to abstain from caffeine, smoking, alcohol, and taking vasoactive medication for at least 12 h before this assessment.

#### Central pressure waveforms

Central arterial BP and wave reflection were assessed using a SphygmoCor pulse wave analysis system (AtCor Medical, Sydney, Australia). The probe was placed at the site of the strongest radial artery pulse to record a stable pulse wave. After 20 sequential waveforms were acquired, a validated, generalized transfer function was used to generate the corresponding central aortic pressure waveform. The central blood pressures (e.g., cSBP, cDBP, central pulse pressure [cPP]) were automatically calculated. Augmentation indices of the central waveform were measured as indices of wave reflection; AIx (a composite measure of arterial stiffness and wave reflection) was defined as augmented pressure divided by pulse pressure and expressed as a percentage [13]–[14]. To take into account the potential effect of heart rate on AIx, an index normalized for the heart rate of 75 bpm was synchronously analyzed.

#### Arterial stiffness

After participants rested in the supine position for 5 to 10 min, PWV was determined using a Complior SP device (Artech Medical, France), which allows online pulse wave recording and automatic calculation of PWV. Two transducers were used: one positioned at the base of the neck over the common carotid artery, and the other over the femoral artery. Two different pulse waves were obtained simultaneously at two sites, the measurement was repeated over 10 different cardiac cycles, and the mean value was used for the final analysis. PWV was calculated from the pulse transit time and distance traveled by the pulse between the two recording sites (measured on the surface of the body in meters), according to the following formula: PWV (m/s) = distance (m)/transit time (s) [15]. Carotid-femoral PWV is a well-established index of aortic arterial stiffness [16].

### Definition of Variables

Essential hypertension was defined as (i) systolic blood pressure (SBP) ≥140 mmHg, and/or (ii) diastolic blood pressure (DBP) ≥90 mmHg, and/or (iii) self-reported use of antihypertensive medication [17].

A subject with any of the following was considered to have DM: (i) fasting venous blood glucose ≥7.0 mmol/l, (ii) 2-h plasma glucose ≥11.1 mmol/l during an OGTT, (iii) symptoms of hyperglycemia and casual plasma glucose ≥11.1 mmol/l, or (iv) the subject was taking antihyperglycemic medication [18].

### Statistical Analysis

Results are expressed as percentages for dichotomous variables, and mean±SD or median (interquartile range) for continuous variables; tHcy level and other biomarkers were normalized by natural logarithm transformation, as necessary. Plasma tHcy levels were categorized as quartile 1 (≤14.0 µmol/l, n = 420), quartile 2 (14.1–17.1 µmol/l, n = 420), quartile 3 (17.2–21.5 µmol/l, n = 420), and quartile 4 (≥21.6 µmol/l, n = 420). Quartiles 1 to 3 were defined as low tHcy (<21.6 µmol/l), and quartile 4 was defined as high tHcy (≥21.6 µmol/l). Arterial properties included in the analyses were brachial BP, central arterial BP, cf-PWV, and heart rate-corrected AIx. Subjects were classified into four groups according to BP (hypertensive vs. normotensive) and tHcy level (<21.6 vs. ≥21.6 µmol/l).

The four groups were compared by one-way ANOVA test (continuous variables) and chi-square test (categorical variables). A general linear model univariate analysis post hoc multiple comparison with Bonferroni’s correction was used to compare cf-PWV and AIx among the groups after adjustment for covariates. Pearson and partial correlation coefficients were used to determine the relationship between tHcy and BP in normotensive and hypertensive subjects after adjusting for age, gender, body mass index, ratio of total to high-density lipoprotein (HDL) cholesterol, blood glucose, current cigarette smoking, eGFR, and prior diagnosis of CVD. Independent determinants of cf-PWV and central AIx were identified by multiple linear stepwise regression analysis (dependent variables: cf-PWV or central AIx; independent variables: age, gender, MAP, height, weight, waist-to-hip ratio, heart rate, current cigarette smoking, antihypertensive medication use, total cholesterol, HDL cholesterol, triglycerides, blood glucose, eGFR, and tHcy levels).

All analyses were conducted using SPSS software for Windows, version 13.0J (SPSS, Chicago, IL, USA). P values <0.05 were considered statistical significant.

## Results

### Clinical Characteristics of Subjects Categorized by BP and tHcy Level

Of the 1680 subjects included in the analysis, 709 were male (42.2%), and the mean age was 61.55±10.90 years (range 24–96 years). Of these, 847 had hypertension (50.4%), 362 had DM (21.5%), and 420 were current smokers (25.0%). The median value of plasma tHcy concentration was 17.2 µmol/l.

Participants were divided into four groups based on blood pressure and plasma tHcy level: 672 were normotensive with low tHcy (<21.6 µmol/l), 161 were normotensive and high tHcy (≥21.6 µmol/l), 587 were hypertensive with low tHcy, and 260 were hypertensive with high tHcy (Table 1). All CVD risk factors other than total cholesterol and low-density lipoprotein (LDL) cholesterol levels differed significantly among the four groups.

**Table 1:** Clinical characteristics of study participants according to blood pressure and tHcy level.

	Normotension		Hypertension		
Characteristics	Low tHcy	High tHcy	Low tHcy	High tHcy	p
No. of subjects	672	161	587	260	
Age (years)	57.77±10.76	62.27±10.81	63.40±9.53	66.92±9.63	<0.001
Males [n (%)]	216 (32.14)	106 (65.84)	227 (38.67)	160 (61.54)	<0.001
Height (cm)	161.71±7.64	164.52±8.46	161.19±8.36	163.89±9.25	<0.001
Weight (Kg)	64.13±10.42	68.34±11.58	67.96±11.11	71.70±11.59	<0.001
Body mass index (kg/m^2^)	24.48±3.31	25.19±3.62	26.14±3.54	26.67±3.59	<0.001
Waist-hip ratio	0.86±0.06	0.87±0.06	0.88±0.06	0.90±0.06	<0.001
Current smokers [n (%)]	136 (20.24)	51 (31.68)	144 (24.53)	89 (34.23)	<0.001
Total cholesterol (mmol/l)	5.05±0.89	4.95±0.85	5.11±0.95	5.02±0.95	0.178
HDL-cholesterol (mmol/l)	1.45±0.38	1.35±0.36	1.35±0.34	1.29±0.31	<0.001
LDL-cholesterol (mmol/l)	2.94±0.73	2.90±0.69	3.03±0.75	3.02±0.73	0.056
Triglycerides (mmol/l)	1.65±1.13	1.76±1.32	1.92±1.21	1.97±1.35	<0.001
FBG (mmol/l)	5.37±1.68	5.16±1.32	5.62±1.80	5.40±1.48	0.003
2-hPBG (mmol/l)	7.30±3.92	7.22±3.75	8.71±4.08	8.02±3.93	<0.001
eGFR (ml/min/1.73m^2^)	108.03±26.99	97.13±28.43	105.80±27.82	95.68±26.81	<0.001
Uric acid (µmol/l)	273.07±68.90	304.57±72.46	296.79±70.71	325.68±74.41	<0.001
Homocysteine (µmol/l)	15.0 (12.2,17.5)	26.7 (23.3,31.5)	16.1 (13.9,18.5)	26.2 (23.4,30.9)	<0.001
History of CVD [n (%)]	63 (9.37)	23 (14.29)	113 (19.25)	61 (23.46)	<0.001
Diabetes [n (%)]	104 (15.48)	30 (18.63)	162 (27.60)	66 (25.38)	<0.001
Antihypertensive drugs					
CCB [n (%)]	–	–	169 (28.79)	104 (40.00)	<0.05
Diuretics [n (%)]	–	–	71 (12.09)	34 (13.08)	0.462
Beta-blockers [n (%)]	–	–	118 (20.10)	76 (29.23)	<0.05
ACEI [n (%)]	–	–	85 (14.48)	45 (17.31)	0.349
ARB [n (%)]	–	–	54 (9.20)	29 (11.15)	0.657

Continuous variables are expressed as mean (±SD) or median (interquartile range), and categorical variables are expressed as counts and percentages. Low tHcy was defined as tHcy concentration <21.6 µmol/l; high tHcy was defined as tHcy concentration ≥21.6 µmol/l.

HDL: high-density lipoprotein; LDL: low-density lipoprotein; FBG: fasting blood glucose; 2-h PBG: 2-h postprandial blood glucose; eGFR: estimated glomerular filtration rate; CVD: cardiovascular disease; CCB: calcium channel blocker; ACEI: angiotensin-converting enzyme inhibitor; ARB: angiotensin II receptor blocker.

### Influence of tHcy on Central Arterial BPs and Arterial Stiffness

Peripheral blood pressures (SBP, DBP, PP, MAP) and central arterial blood pressures (cSBP, cDBP, cPP, PP amplification) did not differ significantly according to tHcy level among either normotensive or hypertensive subjects (Table 2). Among normotensive subjects, cf-PWV was lower in individuals with low tHcy levels than in those with high tHcy levels; however, this difference was not significant after adjusting for confounding factors. We observed differences in cf-PWV according to plasma tHcy level only among hypertensive subjects. Central AIx did not differ according to tHcy level in either normotensive or hypertensive individuals, and this result remained even after adjusting for confounding factors.

**Table 2:** Peripheral and central blood pressure values and arterial stiffness according to blood pressure and tHcy level.

	Normotension				Hypertension			
Variable	Low tHcy	High tHcy	Crude P	Corrected P	Low tHcy	High tHcy	Crude P	Corrected P
Brachial SBP (mmHg)	120.45±10.91	122.12±11.94	0.076	0.304	142.40±14.88	144.48±15.49	0.063	0.252
Brachial DBP (mmHg)	72.77±7.84	72.82±7.79	0.934	1	79.88±10.61	80.48±10.71	0.447	1
Brachial PP (mmHg)	47.68±9.31	49.29±10.44	0.067	0.268	62.52±13.90	64.01±14.64	0.078	0.312
Brachial MAP (mmHg)	88.66±7.83	89.32±7.99	0.372	1	100.72±10.28	101.88±11.31	0.116	0.464
Heart rate (bpm)	75.41±9.42	74.40±9.37	0.219	0.876	75.63±10.62	76.02±10.14	0.624	1
Central SBP (mmHg)	110.65±12.11	112.22±11.62	0.187	0.748	130.67±14.81	130.48±14.31	0.879	1
Central DBP (mmHg)	72.75±9.56	72.54±8.77	0.818	1	80.09±12.14	79.74±13.14	0.690	1
Central PP (mmHg)	38.17±9.10	40.03±10.40	0.058	0.232	50.45±14.32	50.69±13.68	0.810	1
PP amplification(%)	128.69±22.82	127.08±23.32	0.417	1	128.42±25.53	130.94±26.07	0.179	0.716
cf-PWV (s/m)	10.47±2.55	11.16±2.47	0.007	0.028	12.34±2.84	13.12±3.18	0.001	0.004
Central AIx P75 (%)	26.20±9.78	25.13±9.06	0.192	0.768	27.96±9.53	26.28±10.12	0.052	0.208
cf-PWV*(s/m)	10.83±2.04	10.95±2.16	0.473	1	12.18±2.29	13.37±2.43	<0.001	<0.001
Central AIx P75** (%)	25.86±7.22	26.54±6.98	0.289	1	28.24±7.61	27.17±7.34	0.095	0.38

SBP: systolic blood pressure; DBP: diastolic blood pressure; PP: pulse pressure; MAP: mean arterial blood pressure; cf-PWV: carotid-femoral pulse wave velocity; central AIx P75: central augmentation index corrected for a heart rate of 75 bpm. Low tHcy was defined as tHcy <21.6 µmol/l; high tHcy was defined as tHcy ≥21.6 µmol/l. PP amplification was calculated as the peripheral/central pulse pressure ratio.

* After adjustment for age, gender, heart rate, MAP, blood glucose, and current smoking; **after adjustment for age, gender, body mass index, MAP, blood glucose, and current smoking.

Crude P for difference between strata (High tHcy versus Low tHcy). Corrected P value was obtained by Bonferroni’s correction for multiple testing. In a one-way ANOVA involving 4 group means, there are 4 pairwise comparisons. Thus, Corrected P value =  Crude P Value*4.

### Relationship between Plasma tHcy and BP Parameters

In bivariate analyses, correlations between plasma tHcy and BP parameters were significant only among normotensive subjects; no statistically significant correlations were observed among hypertensive individuals (Table 3). In normotensive subjects, tHcy concentration (natural logarithm-transformed) correlated with brachial SBP (r = 0.109, P<0.001), brachial PP (r = 0.110, P = 0.001), central SBP (r = 0.076, P = 0.025) and central PP (r = 0.125, P<0.001). However, partial correlation analysis revealed that these correlations were not significant after adjusting for other risk factors known to influence tHcy level. No significant association between tHcy concentration and BP parameters was observed in the study population.

**Table 3:** Univariate and partial correlation analyses of tHcy levels and blood pressures.

	Ln(tHcy ) in normotension		Ln(tHcy ) in hypertension	
Correlation	r	R	r	R
Brachial systolic BP	0.109**	−0.031	0.056	0.025
Brachial diastolic BP	0.020	0.025	−0.016	0.052
Brachial pulse pressure	0.110**	−0.058	0.063	−0.008
Brachial MAP	0.065	0.002	0.016	0.045
Central systolic BP	0.076*	0.017	−0.018	−0.026
Central diastolic BP	−0.028	0.008	−0.057	0.004
Central pulse pressure	0.125**	0.022	0.032	−0.026
Pulse pressure amplification	−0.039	−0.077	0.017	0.001

r: Pearson correlation coefficient; R: partial correlation coefficient. **P<0.01; *P<0.05. BP: blood pressure; MAP: mean arterial blood pressure; Ln(tHcy): natural logarithm-transformed plasma total homocysteine.

Partial correlation analyses were performed after adjustment for age, gender, body mass index, ratio of total cholesterol to high-density lipoprotein cholesterol, blood glucose, current smoking, eGFR, and prior diagnosis of CVD.

### Multivariate Analysis of the Relationships between tHcy and cf-PWV and AIx

To determine independent predictors of cf-PWV, multiple regression analysis was performed using the stepwise procedure (Table 4). The analysis showed that for hypertensive subjects tHcy was an independent determinant of cf-PWV; other parameters entered in the model were age, MAP, fasting blood glucose, HDL cholesterol, and height. However, for normotensive subjects the independent predictors of cf-PWV were age, MAP, fasting blood glucose, HDL cholesterol, height, and heart rate, plasma tHcy did not enter the model.

**Table 4:** Independent determinants of cf-PWV and AIx in hypertensive and normotensive subjects.

	cf-PWV			Central AIx			
variables	β	SE	P	variables	β	SE	P
*Hypertensive subjects (R^2^ = 0.519)*				*Hypertensive subjects (R^2^ = 0.423)*			
Age	0.130	0.010	<0.001	MAP	0.241	0.033	<0.001
MAP	0.025	0.009	<0.001	Female	4.015	0.834	<0.001
Ln(tHcy)	0.713	0.247	0.004	Heart rate	−0.418	0.033	<0.001
FBG	0.083	0.020	<0.001	Height	−0.315	0.047	<0.001
HDL-C	−0.588	0.203	0.013	Weight	−0.181	0.037	<0.001
Height	0.026	0.013	0.036	*Normotensive subjects* (*R^2^* = *0.457*)			
*Normotensive subjects (R^2^* = *0.436*)				Age	0.135	0.030	<0.001
Age	0.107	0.006	<0.001	MAP	0.295	0.043	<0.001
MAP	0.031	0.009	<0.001	Female	5.175	1.012	<0.001
FBG	0.087	0.021	<0.001	Heart rate	−0.507	0.036	<0.001
HDL-C	−0.632	0.192	0.001	Height	−0.369	0.059	<0.001
Height	0.037	0.014	0.038	Weight	−0.182	0.037	0.021
Heart rate	0.030	0.008	<0.001	Smoking	2.524	0.876	0.004

β: regression coefficient; MAP: mean arterial blood pressure; ln(tHcy): natural logarithm-transformed plasma total homocysteine; FBG: fasting blood glucose; HDL-C: high-density lipoprotein cholesterol; cf-PWV: carotid-femoral pulse wave velocity; AIx: augmentation index. β and P values are shown only when P<0.05.

Plasma tHcy was not an independent predictor of central AIx among either normotensive or hypertensive individuals (Table 4). Gender, MAP, heart rate, height, and weight were among the independent predictors of central AIx.

## Discussion

Several important findings emerged from our study evaluating central arterial BP and indices of arterial stiffness in a large community-based sample from China. First, we detected a significant positive association between plasma tHcy and arterial stiffness, measured as cf-PWV (aortic PWV), only among hypertensive subjects. Second, plasma tHcy was not an independent predictor of central AIx, and tHcy concentration was not associated with peripheral or central BP. To the best of our knowledge, this is the first study to include both normotensive and hypertensive individuals to evaluate the relationships between circulating tHcy level and BP and arterial stiffness.

The main finding of this study was that plasma tHcy is positively associated with cf-PWV only in hypertension. This finding suggests a potential role for tHcy in arterial wall remodeling in hypertension, leading to arterial stiffness. PWV is a known marker of arterial stiffness and indicator of vascular damage [19], and cf-PWV is associated with the severity of arteriosclerosis and is a predictor of future CVD events [20]. However, the relationship between tHcy and PWV is controversial [10]–[11]. Our results are in line with some previous studies reporting a positive correlation between tHcy concentration and PWV among individuals at increased risk for CVD, i.e., with DM [21], a high risk to develop hypertension [22]–[23], or end-stage renal disease [24]. This relationship between tHcy level and arterial stiffness indices usually has not observed in healthy individuals [25]–[26] or those at low risk for CVD [11].

The mechanisms underlying the relationship between Hcy and arterial stiffness are not entirely clear but may include endothelial dysfunction [27]–[28], smooth muscle cell proliferation [29], collagen synthesis [30], and deterioration of elastin [31], resulting in impaired arterial compliance. Our observations, together with results of published reports, suggest that tHcy may be not a direct cause of arterial stiffness but contributes to vascular damage after the initial vascular dysfunction has already developed. First, the presence of hypertension or more advanced stage of atherosclerotic disease may make the arterial wall (in particular, the endothelium) more susceptible to the deleterious effect of high plasma tHcy [32]. Second, hypertension is a major link between tHcy and aortic arterial stiffness, suggesting that hypertension may interact with tHcy to produce synergistic effects [33]. Hyperhomocysteinemia appears to increase BP, impair the vasorelaxation activity of endothelial-derived nitric oxide, and accelerate BP-induced oxidative stress on endothelial cells [34]. Tayama et al. [6] found that higher circulating tHcy is associated with increased systemic arterial stiffness, which may enhance BP reactivity to stress in hypertensive patients. The mechanical effects of high BP and the toxic effects of tHcy on the endothelium may trigger the ‘‘response to injury’’ phenomenon [3].

The second important finding of this study is that tHcy was not independently associated with central AIx in hypertension or normotension. These results are consistent with those reported by the B-PROOF study [35], which found that Hcy was associated with aortic PWV but not AIx in elderly individuals. This lack of relationship between tHcy and AIx may be explained by the fact that pressure wave reflections are generated primarily from arterioles [36], suggesting that Hcy does not affect the walls of small arteries. Furthermore, numerous factors other than arterial stiffness influence the height of the reflected wave [37], including physiologic factors such as gender, height, and heart rate and pathological factors such as age, BP, smoking, and medication [38]–[39]. These factors should be taken into account when using AIx as a marker of arterial stiffness. Moreover, although the progression of atherosclerosis stiffen the aortic wall, it does not affect the central AIx [40], and the ability of AIx to assess wave reflection in normotensive healthy individuals is limited [41]. AIx may be a more sensitive marker of arterial stiffness and CVD risk in younger individuals [42].

Finally, this study did not detect an association between tHcy concentration and peripheral or central BP. The association of tHcy levels with high BP has been reported in some but not all prior studies. The Framingham Heart Study did not find a relationship between baseline tHcy with hypertension incidence or with longitudinal blood pressure progression [43]. Eikelboom et al. [44] reported similar conclusions in a case-control study; however, Nygard and colleagues [45] found a weak association between higher tHcy levels and higher DBP in a sample of >12000 men and women from western Norway. However, that study did not report the relationship between SBP and tHcy, and the association between tHcy and DBP was confined to individuals 40 to 42 years of age. The Third National Health and Nutrition Examination Survey (n = 5978) also found a modest association between tHcy and higher DBP and SBP (0.5–1.2 mmHg per 1-SD increment in tHcy) [46]. These discrepancies may be attributed to several factors. First, there were differences in study populations. Our study evaluated community-based population from Beijing consisting of older individuals with more CVD risk factors. Second, most previously published studies focused on the relationship between high tHcy levels and great risk for hypertension [2], [46], [47], whereas few studies estimated the strength of the association between tHcy and BP throughout its continuous range [48]. Third, most studies used only brachial BP as the BP parameter, whereas our study evaluated both peripheral and central BP.

There are several potential limitations of our study. First, all participants were from Beijing; therefore, conclusions drawn from our study cannot be generalized to other ethnic groups. Second, because of the cross-sectional design of our study, we have no direct evidence for a cause–effect relationship. The role of elevated tHcy in increased aortic stiffness requires further investigation by interventional prospective studies. Third, the multiple comparisons may increase the likelihood of type I error. To address this limitation, Bonferroni procedure was used for correction of multiple testing.

## Conclusion

In conclusion, we found that plasma tHcy level is independently associated with arterial stiffness (i.e., cf-PWV) in hypertensive subjects only. This study raises the possibility that reducing plasma tHcy may decrease arterial stiffness in hypertensive individuals.
